# Glutamate Receptor Homologs in Plants: Functions and Evolutionary Origins

**DOI:** 10.3389/fpls.2012.00235

**Published:** 2012-10-30

**Authors:** Michelle Beth Price, John Jelesko, Sakiko Okumoto

**Affiliations:** ^1^Department of Plant Pathology, Physiology and Weed ScienceVirginia Tech, Blacksburg, VA, USA

**Keywords:** ligand-gated channel, calcium ion, membrane protein, glutamate, amino acids

## Abstract

The plant glutamate-like receptor homologs (GLRs) are homologs of mammalian ionotropic glutamate receptors (iGluRs) which were discovered more than 10 years ago, and are hypothesized to be potential amino acid sensors in plants. Although initial progress on this gene family has been hampered by gene redundancy and technical issues such as gene toxicity; genetic, pharmacological, and electrophysiological approaches are starting to uncover the functions of this protein family. In parallel, there has been tremendous progress in elucidating the structure of animal glutamate receptors (iGluRs), which in turn will help understanding of the molecular mechanisms of plant GLR functions. In this review, we will summarize recent progress on the plant GLRs. Emerging evidence implicates plant GLRs in various biological processes in and beyond N sensing, and implies that there is some overlap in the signaling mechanisms of amino acids between plants and animals. Phylogenetic analysis using iGluRs from metazoans, plants, and bacteria showed that the plant GLRs are no more closely related to metazoan iGluRs as they are to bacterial iGluRs, indicating the separation of plant, other eukaryotic, and bacterial GLRs might have happened as early on as the last universal common ancestor. Structural similarities and differences with animal iGluRs, and the implication thereof, are also discussed.

## Introduction

Nitrogen (N) is quantitatively the most important mineral nutrient, and often the limiting factor for plant growth in the field. The availability of N has a profound short- and long-term effect on plant physiology, which involves developmental reprogramming to maximize N use efficiency. Without a doubt, nitrogen-sensing mechanisms that allow such adjustments are essential for the fitness of plants.

Genome-wide studies on plant responses to amino acids have revealed that a large fraction of N-regulated genes (∼81%) require the incorporation of inorganic nitrogen to organic nitrogen, strongly suggesting that plants possess sensory mechanisms for organic N (Gutierrez et al., 2008). Amino acids, formed as the result of the assimilation of inorganic N, serve as N signaling molecules in other organisms, and are considered the prime candidate for organic N signals in plants. Ample evidence demonstrates that amino acid levels affect the activities of key players in nitrogen assimilation pathway through transcriptional and post-transcriptional processes. For example, gene expression of both cytosolic and plastidic glutamine synthetases, GLN1 and GLN2, are regulated by the levels of amino acids in *Arabidopsis* and tobacco (Vincentz et al., 1993; Oliveira et al., 2001; Fritz et al., 2006; Sulieman et al., 2010). The non-protein amino acid GABA (γ-amino-butyric acid), when supplied to plant growth medium, is capable of modulating not only the activity of key enzymes in nitrogen assimilation, but also the uptake of nitrate itself (Barbosa et al., 2010). Furthermore, amino acids are capable of modulating uptake of inorganic and organic nitrogen (Rawat et al., 1999; Nazoa et al., 2003; Hirner et al., 2006). In addition to gene regulation at the transcriptional level, amino acids have been shown to trigger rapid responses when supplied externally to plant cells. For example, exogenous application of amino acids to plants causes a transient cytosolic calcium increase and membrane depolarization (Dennison and Spalding, 2000; Dubos et al., 2003; Demidchik et al., 2004; Qi et al., 2006; Stephens et al., 2008). Further, GABA and d-Ser, have been shown to trigger transient changes in cytosolic [Ca^2+^] in pollen grains (Yu et al., 2006; Michard et al., 2011). These studies suggest that plants have endogenous mechanisms of monitoring the concentration of amino acid levels, enabling modulation of nitrogen metabolism.

Some mechanisms of amino acid sensing have been documented in bacteria, yeast, and mammals. For example, PII proteins found in bacteria and archaea play a pivotal role as master regulators of carbon/nitrogen homeostasis. The conformations of PII proteins are reciprocally regulated by 2-oxoglutarate and glutamine, which signal for carbon and nitrogen abundance, respectively (Leigh and Dodsworth, 2007). In yeast, multiple sensory systems for amino acids have been discovered, namely amino acid permeases such as SSY1 and GAP1 which sense extracellular amino acids (Didion et al., 1998; Wipf et al., 2002; Hundal and Taylor, 2009) and the amino acid-regulated protein kinase that responds to intracellular amino acids (reviewed in (Hinnebusch, 2005). Perception of amino acids by these sensors initiates multiple signaling cascades in which the target of rapamycin (TOR) pathway plays a central role (Zuo et al., 1997; Jacinto and Hall, 2003; Kang et al., 2006). Perception of extracellular amino acids through membrane transport and control of the TOR pathway have also been reported in *Drosophila* (PATH and Slimfast) and mammals (SNAT2; Hundal and Taylor, 2009).

Aside from their roles as nutritional cues, extracellular amino acids play fundamental roles in the signal transduction in the central nervous system of animals. In these tissues, amino acids released from the presynaptic terminal are recognized by membrane receptors on the postsynaptic membrane. Binding of amino acids to these receptors induces opening of amino acid-gated channels or activates G-protein coupled receptors (Kandel et al., 2000).

The mechanisms of amino acid and internal nitrogen level sensing utilized by plants are largely unknown. Sequencing of the model plant *Arabidopsis* genome allowed for identification of proteins that are homologous to the ones involved in amino acid sensing in other organisms. For example, the *Arabidopsis* PII protein homolog GLB1 interacts with and regulates the activity of two enzymes (*N*-acetyl glutamate kinase and acetyl-CoA carboxylase) involved in the regulation of C/N metabolism (Karakas et al., 2011; Kumar et al., 2011). Although GLB1 is unlikely to be the “master regulator” of growth as is its bacterial counterpart, it does seem to be responsible for a sub-network of nitrogen-sensing. It was also found that *Arabidopsis* (and probably other plants) carry genes required for the operation of the TOR pathway (Deprost et al., 2007; Sobolevsky et al., 2009). The depletion of protein phosphatase 2A activity that functions downstream of TOR leads to autophagy and N remobilization, suggesting that the TOR pathway is involved in nutrient signaling in plants (Li et al., 2006). In addition, the possibility of amino acid transporters functioning as amino acid sensors, as is the case with yeast proteins SSY1 and GAP1, has also been suggested although no experimental evidence so far has supported such a role (Tegeder, 2012).

While these pathways are involved in some aspects of N sensing in plants, it is likely that there are additional mechanisms for amino acid sensing. In particular, the mechanism through which amino acids induce rapid signal transduction events such as Ca^2+^ transient and membrane depolarization is largely unknown. Genome sequencing projects of *Arabidopsis* and other plants, including basic land plants such as Bryophytes and Lycophytes, revealed that plants have glutamate-like receptor homologs (GLRs) of mammalian ionotropic glutamate receptors (iGluRs), which are involved in neurotransmission.

Previous studies indicated the involvement of GLRs in various biological processes, such as C/N balance (Kang and Turano, 2003), photosynthesis (Teardo et al., 2010, 2011), responses to abiotic stress (Kang et al., 2004; Meyerhoff et al., 2005), root morphogenesis (Li et al., 2006; Miller et al., 2010), plant-pathogen interaction (Kang et al., 2006; Kwaaitaal et al., 2011), regulation of cellular Ca^2+^ kinetics (Kim et al., 2001; Dubos et al., 2003; Kang et al., 2006; Qi et al., 2006; Cho et al., 2009; Vincill et al., 2012), and pollen tube growth (Michard et al., 2011). While these studies used genetic and pharmacological approaches to study the functions of plant GLRs, the evidence that the plant GLRs function in a similar manner as the mammalian counterpart had been lacking. In particular, ligand-gated activity of plant GLRs had not been demonstrated. Recent work by the Spalding group has, for the first time, demonstrated that at least one plant GLR (AtGLR3.4) is indeed an amino acid-gated channel capable of inducing cytosolic calcium peaks (Vincill et al., 2012). This finding implies that GLRs are indeed capable of perceiving and transducing amino acid signals. During the same period, the structures of animal iGluR have been well characterized. Now we know the structural basis of ligand binding, the interaction between amino terminal domains (ATD), and the molecular structure of the channel domain for animal iGluRs (Mayer, 2011). Since the domain structures of GLRs seem to be well conserved, such structural information is expected to guide the research of plant GLRs.

In this review, we will summarize the recent progress in understanding the function of plant GLRs. Although the picture is far from being complete, critical channel properties of GLRs are starting to be elucidated. Information gained about structural components on animal iGluRs and the implications to plant GLRs are summarized. In addition, phylogenetic relationships between plant GLRs and iGluRs from other organisms have also been discussed.

## The Structures of Glutamate Receptors

### Secondary and tertiary structures of glutamate receptors

Mammalian ionotropic GluRs are classified into four classes based on their pharmacological response to agonists and antagonists: α-amino-3-hydroxy-5-methyl-4-isoxazole propionate (AMPA), kainate (KA), and *N*-methyl-d-aspartate (NMDA) and δ (no known ligands; Mayer, 2005; Connaughton, 2007). These classifications are not rigid however, as a result of sequence similarity and cross-reactivity between classes, often resulting in functional grouping of AMPA, δ, and KA together as the non-NMDA receptors (Kandel et al., 2000; Connaughton, 2007). Paralogs of iGluRs can be found in bacteria, metazoans, and plants. They share the basic structure of domains constituting the ligand binding site and trans-membrane domains, but there is a significant difference in the structures that may have implications in the function of these channels.

**Figure 1:**
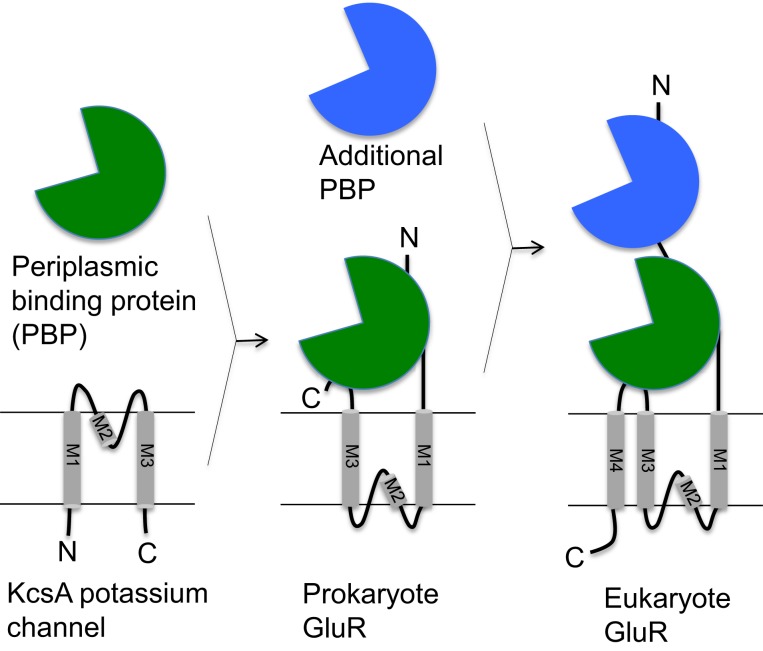
**Schematic representations of prokaryote and eukaryote glutamate receptors**. The proposed origins of ligand binding domain (LBD, green) and amino terminal domain (ATD, blue), and the channel domain. Bacterial periplasmic binding protein and potassium channel are shown for comparison. Proposed gene fusion events that gave rise to the eukaryotic glutamate receptors are represented by arrows.

The minimal structure of an ionotropic glutamate receptor consists of a ligand binding domain (LBD) and a channel-forming domain (Figure 1). A LBD consists of two subdomains, GlnH1/S1, and GlnH2/S2, which are considered to have evolved from periplasmic binding proteins of bacteria because of significant primary sequence similarity (Nakanishi et al., 1990). Indeed, crystal structures of LBDs of all iGluRs analyzed so far has revealed striking structural similarities between the LBDs and bacterial glutamine binding protein (Armstrong et al., 1998; Armstrong and Gouaux, 2000; Naur et al., 2005). The channel-forming domain consists of two or three complete trans-membrane domains (M1 and M3 in prokaryotic channels; M1, M3, and M4 in eukaryotic channels) and one partial trans-membrane domain (M2) that forms a pore-loop (P-loop) structure (Kandel et al., 2000). The structure formed by the M1, M3, and P-loop resembles the structure of tetrameric potassium channels such as KcsA, with inverted topology (MacKinnon, 2003). Due to these structural similarities, prokaryotic iGluRs are considered to be the result of a fusion between bacterial periplasmic binding proteins (PBP) and potassium channels.

**Figure 2:**
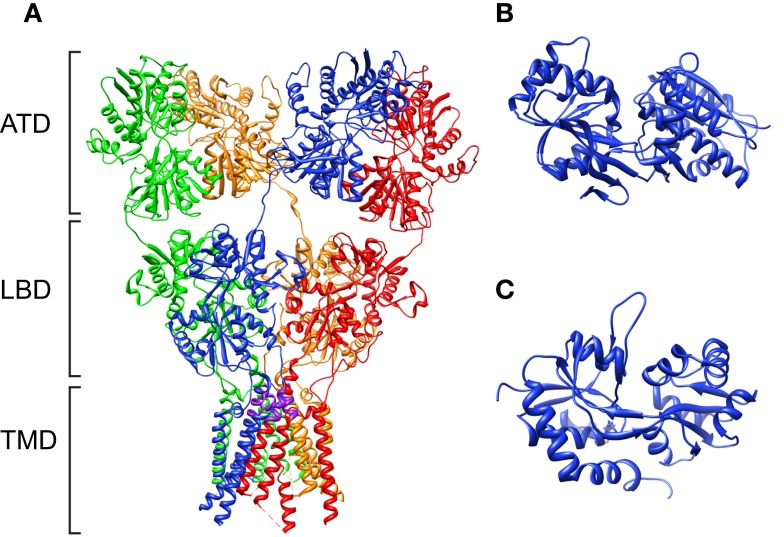
**The crystal structure of the rat glutamate receptor, GluA2**. **(A)** Structure of the intact channel (3KG2; Sobolevsky et al., 2009). Four subunits are shown in different colors. The “SYTANLAA” motif, which consists of the narrowest part of the channel in each subunit, is marked in purple. **(B,C)** The structures of isolated ATD (3H5V; Jin et al., 2009) and LBD (2UXA; Greger et al., 2006), respectively. Note the “venus-flytrap” like structure in both domains.

While bacterial iGluRs consist only of the LBD and the channel-forming domain (Chen et al., 1999), eukaryotic iGluRs possess an additional ATD. Similar to LBD, ATD share sequence and structural similarity with bacterial periplasmic binding proteins, presumably incorporated through another fusion event (Figure 2). The ATDs are responsible for the interaction between the subunits (see Structure and Subunit Compositions of Glutamate Receptors), which in turn contributes to determining the subunit composition of the channel (Jin et al., 2009). Further, the ATD of NMDA receptors can bind a wide range of molecules and ions that work as modulators of the channel activities (Lipton et al., 1997).

Plant iGluRs share the signature “three plus one” trans-membrane domains M1 to M4 as well as the putative LBDs GlnH1 and GlnH2, which show high amino acid sequence identity (63–16%), particularly with the M3 domain (63%) with animal NMDA receptor iGluRs (Lam et al., 1998; Chiu et al., 1999). In addition, the predicted membrane topology and orientation of the protein, with the LBDs exposed to the external side of the membrane, are considered to be conserved (Lam et al., 1998; Dubos et al., 2003, 2005; Furukawa et al., 2005).

### Structure and subunit compositions of glutamate receptors

Early evidence favored a pentameric structure for iGluRs based on the sizes of chemically cross-linked proteins and the number of distinct channel activities produced by the mixture of two subunits (Dingledine et al., 1999). However, an overwhelming number of studies analyzing structures, desensitization properties and cross-linking between subunits through cysteines now suggest that mammalian iGluRs assemble as tetramers (reviewed in Mayer, 2006; Traynelis et al., 2010). In mammals, functional ligand-gated channels can be formed from either homo- or heteromers of four subunits within the same agonist class (Rosenmund et al., 1998). NMDA receptors form obligatory hetero-tetramers consisting of two glycine-binding subunits and glutamate-binding subunits (Monyer et al., 1992), whereas some AMPA and kainate receptors can form functional homo-tetramers (Mano and Teichberg, 1998). The subunit composition dictates the functional properties of the channel, resulting in a large number of receptor types which function differently *in vivo* (Mayer, 2005). In addition, alternative splicing and RNA editing of iGluRs further increases the diversity of the receptor complexes (Egebjerg and Heinemann, 1993; Gereau and Swanson, 2008).

In 2009, the crystal structure of the homo-tetrameric AMPA receptor, rat GluA2 was resolved, shedding light onto the assembly of the entire channel (Sobolevsky et al., 2009). The resolved crystal structure has a “Y”-shape, where the ATD and LBD spread outward from the more compact channel-forming domains (Figure 2). The tetramer is formed as “dimer of dimers,” and the ATD and LBD exhibits approximate overall twofold molecular symmetry to the axis perpendicular to the membrane. The trans-membrane domain, on the other hand, assumes a fourfold rotational symmetry that is remarkably similar to the bacterial potassium channel KcsA (Doyle et al., 1998).

The structure revealed extensive inter-subunit interaction through ATDs (interface ∼330 Å^2^), which was essentially identical to what was observed in the crystal structures of isolated ATDs (Sobolevsky et al., 2009; Mayer, 2011). On the other hand, the inter-subunit interaction in LBDs is much smaller (interface ∼224 Å^2^), hence the role of LBD in the subunit assembly is considered to be minimal. This result corroborates the previous studies using isolated ATD domains: ATDs of two interacting subunits exhibit very high affinity (e.g., 11 nM for GluR6 and KA2 heterodimer, 0.7 μM for NR1 and NR2 heterodimer) to each other compared to the affinity for itself (Karakas et al., 2011; Kumar et al., 2011). Therefore, while ATD might not be the only domain that dictates the interaction partners (Pasternack et al., 2002), it is considered to play an important role in the correct assembly of subunits.

The subunit compositions of plant iGluRs are unknown. Co-expression analysis using single-cell sampling revealed that, at least in *Arabidopsis* leaf epidermal and mesophyll cells, there are five to six GLRs co-expressed on average, therefore hetero-tetramer formation is quite likely (Roy et al., 2008). In studies using T-DNA insertion mutants of AtGLR3.3 and 3.4, it was shown that the response to all six amino acids that can induce membrane depolarization (Ala, Cys, Asn, Glu, Ser, Gly) were affected in *glr3.3* mutants, while in *glr3.4* mutants responses were affected in only a subset of amino acids (Stephens et al., 2008). These results support a model where GLR3.3 is included in all receptor complexes in the cell type tested (hypocotyl) whereas a sub-fraction of complexes include at least GLR3.3 and 3.4. To date, heterologous expression in mammalian cells proved that AtGLR3.4 can form a homo-meric channel (Vincill et al., 2012). Further investigations are necessary to understand the subunit compositions of GLRs *in vivo*.

## Evolutionary Origin of Plant Glutamate Receptors

The 20 *At*GLRs have been divided into three distinct phylogenetic clades on the basis of parsimony analysis with bacterial amino acid binding proteins as out-groups (Chiu et al., 1999). Examination of amino acid sequence similarity between GLRs and various kinds of ion channels such as animal iGluRs, potassium channels, acetylcholine receptors, and GABA_A_ receptors suggested that the plant GLRs are most closely related to animal iGluRs (Chiu et al., 1999). Phylogenetic analyses using both parsimony and neighbor joining suggests that plant and animal iGluRs diverged from a common ancestor as opposed to convergent evolution of genes with similar structure and function (Chiu et al., 1999).

**Figure 3:**
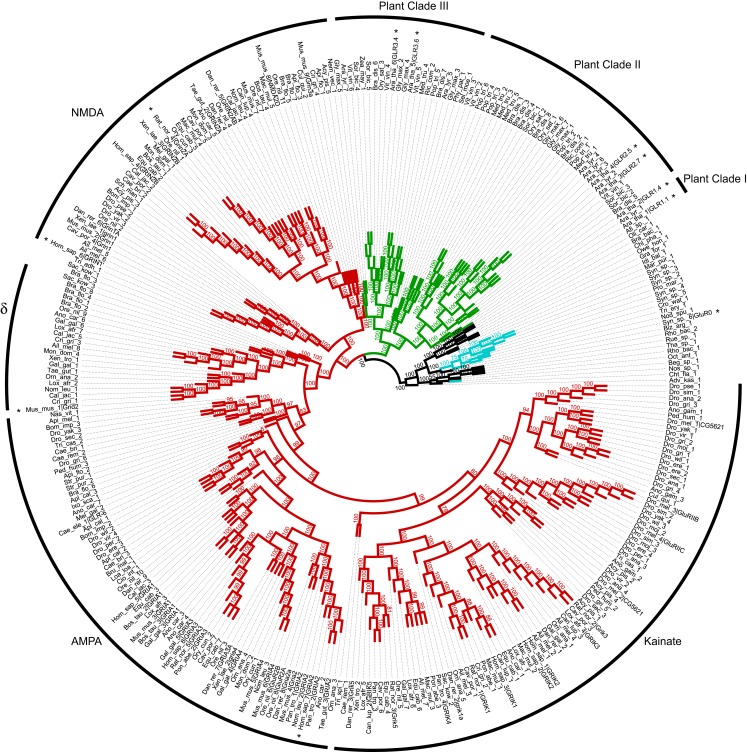
**Phylogenetic tree of plant, animal, and bacterial glutamate receptors**. Metazoan, bacteria, cyanobacteria, plant proteins are marked in red, black, cyan, and green, respectively. NMDA, AMPA, and Kainate subfamilies of mammalian receptors are also indicated. Accession numbers of proteins included in the tree are provided in the Table S2 in Supplemental Material. Sequences represented in Figure 4 are marked with asterisks.

The deep phylogenetic relationships between plants, metazoans, and bacteria GLRs were investigated using statistically oriented phylogenetic methods that are amenable to formal hypothesis testing of alternative molecular evolution models. GLR homologs were identified using the AtGLR1.1 and the human NMDA receptor NR1-3 sequences as queries using a sensitive similarity search algorithm (SSEARCH) enforcing a criteria for establishing homology (i.e., common ancestry) that combined both a maximum expectation value of *e*^−4^ and mutual hits by both query sequences. A bootstrap Maximum Likelihood (ML) phylogenetic analysis indicated a tripartite basal split between bacterial, plant, and other eukaryotic GLR homologs (Figure 3). This topology had good bootstrap support and was generally consistent with the separation of bacteria from eukaryotes in the tree of life (ToL), in agreement with previously published results (Janovjak et al., 2011). Cyanobacterial iGluRs clustered tightly with other bacterial homologs, suggesting against the possibility that the plant iGluRs have a cyanobacterial origin. The hypothesis of a cyanobacterial origin of plant GLRs was also statistically rejected by likelihood ratio testing of phylogenetic analyses using alternatively constrained topologies (Table S1 in Supplemental Material).

The basal positioning of the plant GLR homologs relative to other eukaryotic GLR homologs was also consistent with a very ancient separation of plant GLRs from other eukaryotic GLRs. Indeed, likelihood ratio testing indicated that a model of monophyletic plant-eukaryotic association was not a statistically significant better fit to the data than either monophyletic plant-bacteria association or monophyletic eukaryotic-bacteria association models (Table S1 in Supplemental Material). Thus, plant GLRs were no more closely related to eukaryotic GLR homologs than either plant or eukaryotic GLRs were to the bacterial GLRs. These results indicate a very ancient separation of plant, eukaryotic, and bacterial GLRs that dates back to perhaps as far as the separation of the last universal common ancestor (LUCA) in the ToL, or to the very early evolution of the Eukaryotic domain. This GLR molecular evolutionary model is consistent with the current placement of the Plantae (Archaeplastida) as one of the five super-groups of eukaryote taxonomy (Simpson and Roger, 2002; Adl et al., 2005; Keeling et al., 2005). Thus, the plant GLR homologs should be considered as phylogenetically distinct from metazoan GLRs as they are from the bacterial GLRs.

## Properties of Plant Glutamate Receptors

### Ligands to the glutamate receptors

Mammalian glutamate receptor subunits bind to a number of endogenous substrates, including glutamate, aspartate, glycine, l- and d-serine, and homocysteine (Lipton et al., 1997; Kandel et al., 2000; Schwartz, 2000; Wolosker, 2006). To date, more than 150 high-resolution crystal structures have been obtained for multiple iGluR subtypes. These studies unequivocally showed that the LBD undergoes “venus-flytrap”-like movement when the ligand binds to the cleft between the two lobes. Although the structure of an intact iGluR in the ligand-bound, open state is not available, it is assumed that such conformational change induces opening of the channel. In fact, the potency of an agonist for an iGluR is very well correlated with the degree of domain closure induced by the compound (Pohlsgaard et al., 2011).

**Figure 4:**
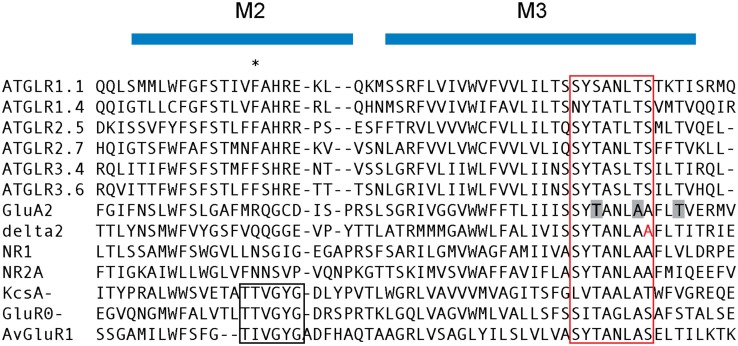
**An alignment of the regions consisting of the selectivity filter and the constrictive domain [the end of M2 (P-loop) and M3 region, respectively]**. The Q/R/N site of mammalian glutamate receptors is marked with an asterisk. The SYTANLAA motif conserved among glutamate receptors is marked in red box. Residues that form the constrictive portion of GluA2 channel is marked with gray. The “lurcher” residue in δ2 is marked in red. The TXVGYG motif in the potassium channel KcsA, GluR0, and AvGluR1 is also marked by a box. AGI and Genbank accession numbers: ATGLR1.1, at3g04110; ATGLR1.4, at3g07520; ATGLR2.5, at5g11210; ATGLR2.7, at2g29120; ATGLR3.4, at1g05200; ATGLR3.6, at3G51480; GluA2 (*Rattus norvegicus*), NP_000817.2; delta2 (*Mus musculus*), *NP_032193.1*; NR1 (*Homo sapiens*), NP_015566.1; NR2A (*Rattus norvegicus*), NP_036705.3; KcsA (*Streptomyces lividans*), P0A334.1; GluR0 (*Synechocystis* sp*. PCC 6803*), ZP_06526299.1; AvGluR1 (*Adineta vaga*), ADW94593.1.

For more than a decade, the ligands for plant GLRs were not known. Initial sequence analysis revealed that plant iGluRs carry a mutation in the pore-forming M3 region which is known to render the mammalian δ2 receptor constitutively active (Zuo et al., 1997; Chiu et al., 1999; Figure 4), suggesting the possibility that plant iGluRs might not function as ligand-gated channels. On the other hand, glutamate and other amino acids are able to induce membrane depolarization and Ca^2+^ conductance in plants, suggesting that there are amino acid-gated calcium channels in plants (Dennison and Spalding, 2000; Dubos et al., 2003; Qi et al., 2006; Stephens et al., 2008). In addition, various studies using agonists and antagonists of mammalian iGluRs indicated that agonists and antagonists that bind to the LBD of animal iGluR are also pharmacologically active in plants (see Properties of Plant Glutamate Receptors).

In a recent study, *At*GLR3.4 was shown to be gated by Asn, Ser, and Gly when expressed in mammalian cells, demonstrating that at least one plant GLRs is capable of forming an amino acid-gated channel (Vincill et al., 2012). This result corroborates with previous work using a structural modeling approach, which indicated Gly could be a more likely agonist than Glu for the majority of GLRs including AtGLR3.4 (Dubos et al., 2003). Although the LBD structures of plant GLRs are not known, it is highly likely that the binding of these amino acids to the LBD of AtGLR3.4 causes opening of the channel. If this is the case, LBDs of plant GLRs have much broader specificity to ligands compared to their animal counterparts. To examine whether the LBD functions in a similar manner as its mammalian counterpart, ligand binding needs to be proven. The molecular structure of AtGLR3.4 in combination with the proposed ligands will help in understanding the basis of ligand gating mechanisms. Ligand specificity of other plant GLRs remains to be investigated.

### Channel selectivity

Mammalian iGluRs are non-selective cation channels (NSCCs) that function to conduct Na^+^, K^+^, and Ca^2+^cations in the presence of glutamate (Kandel et al., 2000; Davenport, 2002; Furukawa et al., 2005). The selectivity for cations is determined by residues in the M2 and M3 regions that line the pore (Panchenko et al., 2001). For example, conversion of a glutamine residue into arginine through RNA editing in GluA2 results in a reduced permeability to Ca^2+^ (Egebjerg and Heinemann, 1993). This so-called Q/R/N site topologically overlaps with the selectivity filter of the potassium channel KcsA and the bacterial glutamate receptor GluR0, reinforcing the role of this domain in the determination of ion permeability. The recently resolved GluA2 structure revealed remarkable similarity between the overall topology between GluA2 and KcsA ion channel domains. The M3 domains of four subunits cross each other at the “SYTANLAA” motif, which is highly conserved in iGluRs, and form the narrowest portion of the channel (Figures 2 and 4; Sobolevsky et al., 2009).

While the “SYTANLAA” motif is also conserved in plant GLRs, primary sequence of the remainder of the M2 and M3 regions, which determines the selectivity of the channel, are completely different from animal iGluRs, hence it is reasonable to expect that the channel specificities would be different (Figure 4; Davenport, 2002). In fact, predicting the selectivity of the channel solely by the primary sequence is not possible, highlighted by a recent discovery of a glutamate receptor channel from *Adineta vaga*, which possesses a potassium selective “TXVGYG” motif, yet is permeable to Na^+^ (Janovjak et al., 2011). Studies conducted thus far suggest that plant GLRs are NSCCs. *Arabidopsis* plants over-expressing the *At*GLR3.2 gene exhibited a phenotype consistent with Ca^2+^ deficiency that was reversed when supplemented with exogenous Ca^2+^ (Kim et al., 2001). These plants also exhibited an increased sensitivity to K^+^, Na^+^, and Mg^2+^cations, consistent with their putative roles as NSCCs (Kim et al., 2001). Additionally, expression of *At*GLR3.7 in *Xenopus* oocytes enhanced plasma membrane conductance of Ba^2+^, Ca^2+^, and Na^+^ ions, providing further evidence that plant GLRs function as NSCCs (Roy et al., 2008). Transplantation of the pore domains of *At*GLR1.1 and 1.4 into rat GluR1 and GluR6 chimeras produced functional K^+^, Na^+^, and Ca^2+^ channels, suggesting that *At*GLR1.1 and 1.4 function as NSCC (Tapken and Hollmann, 2008). One notable exception is *At*GLR3.4, which was shown to be highly selective to Ca^2+^ by whole-cell patch clamp of HEK293 cells expressing *At*GLR3.4 (Vincill et al., 2012).

### Pharmacological properties of plant GLRs

Glutamate-like receptor homologs form a large family in plants, presenting a challenge for genetic approaches due to the potential for functional redundancy. To circumvent this, the utilization of pharmacology to act on multiple members of the family is a common strategy. As discussed previously, the LBD of plant GLRs shares homology at the amino acid level with mammalian iGluRs. Thus, several groups have investigated whether plant GLRs are influenced similarly to their mammalian counterparts in response to known iGluR agonists and antagonists (Lam et al., 1998; Brenner et al., 2000).

The possible *in vivo* function of plant GLRs was first examined by investigating the responses of plants to the known competitive antagonist 6,7-dinitroquinoxaline-2,3-(1*H*,4*H*)-dione (DNQX; Lam et al., 1998). When *Arabidopsis* seedlings were treated with DNQX, plants grown in light exhibited a dose- and light-dependent increase in hypocotyl elongation and reduced light-induced chlorophyll synthesis (Lam et al., 1998). Likewise, when light grown *Arabidopsis* seedlings were treated with *S*(+)-β-methyl-α,β-diaminopropionic acid (BMAA), an agonist of AMPA-kainate iGluRs, and a glutamate analog, hypocotyl elongation was increased while cotyledon opening was impaired (Brenner et al., 2000). BMAA-induced hypocotyl responses are alleviated when exogenous glutamate is applied, suggesting that there may be a conserved mechanism for the activity of BMAA between mammalian and plant iGluRs (Brenner et al., 2000). In addition, the fact that two different compounds capable of interacting with mammalian iGluRs, DNQX, and BMAA, each induce hypocotyl elongation in light grown seedlings suggests a role for *At*GLRs in photomorphogenic development (Brenner et al., 2000). DNQX, along with AP-5 and 6-cyano-7-nitroquinoxaline-2,3-dione (CNQX), was also recently found to suppress pollen tube growth in tobacco (Michard et al., 2011).

In another study, it was revealed that Ca^2+^ conductance induced by microbe-associated molecular patterns (MAMPs) are inhibited specifically by mammalian glutamate receptor agonists (glutamate, aspartate) and antagonists (AP-5, AP7, and kynurenic acid; Kwaaitaal et al., 2011). In this study, DNQX, which had a pharmacological effect on the hypocotyl elongation, was not effective in inducing Ca^2+^ influx, suggesting that a different molecular target (e.g., a different subfamily of GLRs) are responsible for the influx of calcium compared to the situation in the hypocotyl. Direct evidence showing that mammalian glutamate receptor agonists and antagonists do bind plant GLRs, or discovery of more agonists and antagonists of plant GLRs, would accelerate the research tremendously.

### Subcellular localization of plant GLRs

Localization of iGluRs from metazoans is highly dynamic, continuously cycling between endosomal compartments and the “site of action,” the postsynaptic membrane (Moss and Henley, 2002). So far, only two plant GLRs have been characterized for their subcellular localizations. AtGLR3.4 was shown to localize to the plasma membrane (Meyerhoff et al., 2005; Teardo et al., 2010, 2011; Vincill et al., 2012). Likewise, a GFP-fusion of a GLR from small radish (RsGluR) localized to the plasma membrane (Kang et al., 2006). Interestingly though, biochemical analysis using antibodies detected the presence of AtGLR3.4 in the chloroplast in addition to the plasma membrane (Teardo et al., 2011), and similar result was obtained in spinach (Teardo et al., 2010). Whether such dual-localization is common to other members of plant GLRs remains to be investigated.

## Physiological Roles of Plant GLRs

Now that ligand-gated calcium conductance of *At*GLR3.4 has been shown in the heterologous expression system, it seems that there is little doubt about at least one, probably more, of plant GLRs being amino acid-activated channels (Vincill et al., 2012). Although there is no experimental evidence showing that the topology of plant GLRs are identical to the animal GLRs, homology to the well characterized iGluRs from other organisms make such a scenario quite likely. Thus, one function of GLRs would be to sense amino acids at the exterior of the membrane in which the GLRs are localized.

A number of studies suggest that amino acid content in the apoplast is influenced by factors such as carbon and nitrogen supplies and stress. Microarray analysis using an inhibitor of glutamine biosynthesis suggested that a significant fraction of N-responsive genes (126/834) respond to the extracellular glutamate/glutamine, indicating a sensory mechanism for apoplasmic amino acids (Gutierrez et al., 2008). GLRs expressed at the plasma membrane function as sensory mechanisms for apoplasmic amino acids. Antisense plants for *At*GLR1.1 show altered transcript abundance in carbon and nitrogen metabolic enzymes such as cytosolic glutamine synthase (GS1), cytosolic aspartate aminotransferase (AAT2), nitrate reductase (NR1), nitrite reductase (NiR), nitrate transporter (CHL1), and hexokinase (HXK1; Kang and Turano, 2003). These results suggest a role for GLR1.1 in regulation of carbon and nitrogen metabolism (Kang and Turano, 2003; Kang et al., 2004).

In addition to the local nitrogen status, plants have intricate mechanism of communicating nitrogen availability in the rhizosphere to the above-ground organs (Ruffel et al., 2008, 2011). Although molecular mechanisms for such long-distance communication are not completely understood, it is well documented that the feeding of amino acids through the xylem induces transcriptional and post-transcriptional changes in key enzymes of the nitrogen assimilation pathway (Vincentz et al., 1993; Fritz et al., 2006; Sulieman et al., 2010). The amino acid profile in xylem sap, which is a continuum of the apoplasmic space, is influenced by many factors such as nitrogen supply, light cycle, and stress (Rosnitschek-Schimmel, 1985; Lam et al., 1995; Mayer, 2011). It is tempting to speculate that GLRs could be involved in amino acid sensing in the xylem. The expression patterns of GLRs suggest that at least some of them are expressed in the vasculature (Kim et al., 2001; Chiu et al., 2002; Meyerhoff et al., 2005; Cho et al., 2009). Perhaps GLRs play roles in the communication of C/N status in the apoplasm to the cells surrounding vascular tissue, in the form of Ca^2+^ signaling.

Recent studies reporting the localization of GLRs in chloroplasts indicate an additional role of GLRs in this organ. Indeed, plants carrying T-DNA insertions in AtGLR3.4 showed weak photosynthetic phenotypes (Teardo et al., 2010, 2011). The exact roles of GLRs in chloroplasts awaits further investigation.

Amino acids are also involved in host-pathogen interactions. Changes in the amino acid profile in the apoplasm upon pathogen infection have been documented in multiple host-pathogen combinations (Solomon and Oliver, 2001). Recent findings show that the availability of apoplasmic GABA is important for colonization of tomato by *Pseudomonas syringae*, yet a relatively high concentration of GABA enhances the defense response of plants (Park et al., 2010). GABA is also involved in quorum sensing in *Agrobacterium*, which is counteracted by another amino acid, proline (Chevrot et al., 2006; Haudecoeur et al., 2009). Further, it is interesting that the NMDA receptor agonists applied to *Arabidopsis* seedlings inhibit the cytosolic calcium peaks induced by MAMPs (Kwaaitaal et al., 2011). Whether GLRs play a role in sensing changes in amino acids induced by plant-pathogen interaction remains to be seen.

Interestingly, pharmacological and genetic approaches to understand the functions of GLR revealed their roles in biological processes that were previously not linked to extracellular amino acids. Recently it was demonstrated that gene insertions in *At*GLR1.2 and 3.7 result in a pollen tube phenotype, and that the Ca^2+^ signature was altered in a *glr1.2* mutant. Moreover, d-Ser, an agonist to animal iGluR, is capable of inducing calcium peaks in the growing pollen tube, and pollen tube growth is disturbed in a knock-out mutant of serine-racemase (SR1). These results suggested a possible involvement of d-Ser and GLRs in male gametophyte-pistil communication (Michard et al., 2011). Interestingly, a concentration gradient of another amino acid, GABA was shown to be important in the guidance of the pollen tube (Palanivelu et al., 2003). It is possible that more members of the GLR family are involved in such cell-to-cell communication in plants. Likewise, it was shown that *At*GLR3.1 is expressed preferentially in guard cells, and over-expression of *At*GLR3.1 has been shown to lead to the impairment of stomatal closure that is induced by external Ca^2+^ (Cho et al., 2009). Since amino acid-gated channel activity is not reported for *At*GLR3.1, whether the channel conductivity is influenced by apoplasmic amino acid remain to be seen.

## Future Perspectives

After 14 years of research, we are now beginning to understand the diverse functions of plant GLRs. However, our understanding of their molecular mechanisms is still in its infancy. For example, conductivity and ligand spectrum for more subunits need to be elucidated in order to understand their *in vivo* function. Successful expression in heterologous systems such as mammalian cell culture and *Xenopus* oocyte would be a key step. It has previously been reported that plant GLRs do not localize to the membrane when expressed in heterologous systems (Li et al., 2006). Analogous to some of the obligatory heteromers in animal systems, correct formation of heteromer might be necessary for the trafficking of receptor complexes to the plasma membrane (Qiu et al., 2009). Co-expression analysis at a higher resolution, as well as protein–protein interaction studies in a heterologous system such as yeast (Lalonde et al., 2010) will help in identifying the necessary components of functional channels.

Another open area is the post-translational regulation of plant GLRs. The C-termini of animal GLRs contain multiple sites for phosphorylation and protein–protein interaction, which in turn determine the localization and surface expression of the receptor (Chen and Roche, 2007; Bard and Groc, 2011). Interestingly, multiple GLRs (1.2, 2.1, 2.9, 3.4, 3.7) were identified as potential 14-3-3 client proteins in a proteomics study (Chang et al., 2009). The effect of such interactions on channel properties would be an interesting subject.

## Conflict of Interest Statement

The authors declare that the research was conducted in the absence of any commercial or financial relationships that could be construed as a potential conflict of interest.

## Supplementary Material

The Supplementary Material for this article can be found online at: http://www.frontiersin.org/Plant_Traffic_and_Transport/10.3389/fpls.2012.00235/abstract
